# Structural and functional properties of neodymium-doped hydroxyapatite nanoparticles for biomedical applications

**DOI:** 10.1016/j.btre.2025.e00916

**Published:** 2025-08-15

**Authors:** K.Sai Manogna, K. Kusuma, G.Rajasekhara Reddy, B.Deva Prasad Raju, N.John Sushma

**Affiliations:** a Department of Biotechnology, Sri Padmavati Mahila Visvavidyalayam, (Women’s University), Tirupati, 517 502, India; b Department of Physics, Sri Venkateswara University, Tirupati, 517501, India; c School of Mechanical Engineering, Yeungnam University, Gyeongsan, 38541, Republic of Korea

**Keywords:** Anticancer activity, Han: Nd^3+^ NPs, Photoluminescence, CIE chromaticity coordinates, Bioimaging

## Abstract

Neodymium (Nd³⁺) doped hydroxyapatite nanoparticles (Han: Nd³⁺ NPs) were synthesized and systematically characterized to evaluate their structural and functional properties for biomedical applications. X-ray diffraction (XRD) confirmed the crystalline phase retention post-doping, while x-ray photoelectron spectroscopy (XPS) revealed the successful incorporation of Nd³⁺ ions. The doping altered the optical and electronic properties, potentially enhancing bioactivity and imaging capabilities. Preliminary cytotoxicity assessments on MCF7 and 4T1 breast cancer cell lines indicated dose-dependent effects, with IC50 values of 36.13 µg/mL and 64.38 µg/mL, respectively. The study concludes that Han: Nd³⁺ NPs offer promise as multifunctional platforms for cytotoxic response and structural stability, with potential for future application in imaging and targeted breast cancer therapy.

## Highlights

• Doping with Nd³⁺ ions modified the nanoparticles’ optical and structural properties, enhancing their potential for bioimaging and theranostic applications.
• Dose-dependent cytotoxic effects observed on breast cancer cell lines (MCF7 and 4T1), with IC50 values of 36.13 μg/mL and 64.38 μg/mL, indicating potential anticancer activity.
• Potential applications in targeted drug delivery and bioimaging due to their multifunctional properties.

## 1 Introduction

Rare-earth-activated nanoparticles (NPs) have gained prominence in biomedical research due to their exceptional photoluminescence, electronic structure, and energy configurations, making them ideal for bioimaging, theranostics, and optoelectronic applications [1]. Among them, lanthanide-doped nanoparticles (Ln-NPs) exhibit superior photostability, high quantum yield, and long emission lifetimes, enhancing their potential for imaging and therapy [2]. However, precise synthesis and stability control remain challenging [3]. Hydroxyapatite (HA), the primary mineral of bone, serves as an excellent platform for lanthanide doping due to its biocompatibility and structural similarity to native bone [4]. HA nanoparticles (HA-NPs) have been widely explored for biomedical applications, including bone regeneration, drug delivery, and imaging, owing to their tunable surface properties [5]. The incorporation of lanthanide ions into HA matrices enhances their luminescent and targeting properties, making them ideal for bioimaging and drug delivery [6].

Neodymium (Nd³⁺), commonly used in industrial magnets, has shown significant potential in biomedical imaging and theranostics [7,8]. Nd³⁺-doped NPs emit strong near-infrared (NIR) luminescence, allowing deep tissue imaging with minimal photodamage and autofluorescence [9]. Additionally, they have been reported to enhance reactive oxygen species (ROS) generation, selectively inducing cytotoxic effects in cancer cells, highlighting their potential as anticancer agents [10]. Their enhanced permeability and retention (EPR) effect further promotes selective tumor accumulation for imaging and therapy [11]. Traditional imaging modalities like CT, PET, and SPECT face limitations such as low sensitivity and spatial resolution, particularly in early-stage detection [[12], [13], [14], [15]]. To address this, dual-modal fluorescence-based imaging probes have been developed [16]. Organic dyes and quantum dots (QDs) have been explored but suffer from photobleaching, cytotoxicity, and poor biocompatibility [17,18]. In contrast, rare-earth-doped HA NPs, including europium (Eu³⁺) and neodymium (Nd³⁺), offer excellent luminescence, biocompatibility, and stability, making them promising for *in vivo* imaging and targeted drug delivery [19,20].

This study aims to synthesize and characterize luminescent, biocompatible neodymium-doped hydroxyapatite (Nd:HA) nanoparticles via a controlled precipitation method. By integrating Nd³⁺ for near-infrared imaging and ROS-mediated cytotoxicity, this work introduces a multifunctional nanoplatform for cancer theranostics. The physicochemical characteristics, colloidal stability, and biological interactions of the synthesised nanoparticles were methodically assessed. This study presents a streamlined synthesis method utilising tri-sodium citrate to improve pH regulation and nanoparticle distribution alongside comparative cytotoxicity assessments in human (MCF7) and murine (4T1) breast cancer models, contrasting with prior research that typically focuses on imaging or cytotoxicity independently. This dual-model evaluation provides insight into interspecies cytotoxicity variations an aspect often overlooked in similar reports. Together, these features represent a distinct contribution to the development of rare-earth doped hydroxyapatite nanoparticles for theranostic applications.

## 2 Materials and methods

### 2.1 Reagents

Calcium chloride (CaCl_2_), sodium dihydrogen phosphate (NaH_2_PO_4_), trisodium citrate, and disodium hydrogen phosphate (Na_2_HPO_4_) were sourced from Merck, Bangalore, India, and were of analytical grade. Neodymium oxide (Nd_2_O_3_) was obtained from Sigma Aldrich, Bangalore, India, and used for the synthesis of Han: Nd^3+^ NPs. Phosphate buffer solutions were prepared using analytical-grade sodium dihydrogen phosphate and disodium hydrogen phosphate (Merck, India). Doxorubicin, a chemotherapeutic compound, was utilized in our *in vitro* experiments on MCF7 and 4T1 breast cancer cell lines.

### 2.2 Synthesis of neodymium-doped hydroxyapatite nanoparticles

The synthesis proceeded in two main steps: Neodymium chloride (NdCl₃) was prepared by dissolving neodymium oxide (Nd₂O₃) in concentrated hydrochloric acid (HCl), followed by evaporation of the solvent under gentle heating at 70–80 °C until dryness. Nanoparticle synthesis, adapted from previously reported rare-earth element procedures. First, 1.8 g of calcium chloride (CaCl₂) and 0.5 g of NdCl₃ were dissolved in 100 mL of distilled water, and the pH was adjusted to ∼8 using tri-sodium citrate (C₆H₅O₇Na₃). Separately, 10 g of disodium hydrogen phosphate (Na₂HPO₄) was dissolved in 500 mL of water, then added dropwise to the initial solution with continuous stirring for 24 h. The opaque colloidal mixture was filtered, washed with water, decanted, and centrifuged. The resulting pellet was dried and ground into a fine powder, referred to as Han: Nd³⁺ NPs for subsequent analyses.

### 2.3 Particle characterizations

Various advanced techniques were employed to comprehensively characterize the nanoparticles. These techniques included x-ray diffraction spectroscopy (XRD), hydrodynamic size and zeta potential analysis, x-ray photoelectron spectroscopy (XPS), scanning electron microscopy coupled with energy dispersive x-ray spectroscopy (SEM-EDX) and *in vitro* cytotoxicity assessments.

### 2.4 X-ray diffraction spectroscopy (XRD)

Crystalline structures were investigated using XRD data collected with an AERIS PAN analytical diffractometer. Cu Kα1 radiation with a wavelength of 1.5406 Å was employed in para-focusing geometry to optimize diffraction effects. Powdered samples were uniformly positioned on a sample plate to ensure consistency during analysis. The resulting data were processed using high-score software.

### 2.5 Hydrodynamic size and zeta potential analysis

The hydrodynamic diameter and zeta potential of Han: Nd^3+^ NPs were assessed utilizing a dynamic light scattering (DLS) apparatus integrated with a zeta potential analyzer, Malvern Zetasizer Nano ZS. The nanoparticles were suspended in deionized water at a suitable concentration and sonicated for 10 min to achieve uniform dispersion [25]. Hydrodynamic size analysis was conducted at 25 °C and a scattering angle of 173°, with results presented as the Z-average diameter and the polydispersity index (PDI) to indicate size distribution uniformity. Zeta potential tests were conducted in a disposable folding capillary cell at 25 °C to evaluate the surface charge and colloidal stability of the particles [26].

### 2.6 X-ray photoelectron spectroscopy (XPS)

Elemental composition and oxidation states were determined using XPS with the Thermo Scientific K-alpha model. Monochromatic Al Kα X-ray radiation at 1486.6 eV energy was used to determine binding energies of various elements in the Nd^3+^ specimen. Avantage commercial software facilitated data capture and processing, including background correction and noise reduction.

### 2.7 Scanning electron microscopy - energy dispersive x-ray spectroscopy (SEM-EDX)

Microstructural and elemental attributes were analyzed using SEM-EDX. Elemental analysis included carbon, hydrogen, and nitrogen elements, and CHN analysis was conducted using the Perkin Elmer 2400 CHN elemental analyzer. EDX analysis was performed using the EDX APEX system, and a comprehensive solubility profile was constructed for the Neodymium: Nd^3+^ specimen, revealing its interaction dynamics with various solvents.

### 2.8 *In vitro* studies

The cytotoxic potential of Han: Nd³⁺ NPs was assessed using the MTT assay on MCF7 (human breast cancer) and 4T1 (mouse breast cancer) cell lines to assess dose-dependent effects. MCF7 cells were procured from NCCS, Pune, India, while 4T1 cells were obtained from ATCC, USA (Cat No: CRL-2539). MCF7 cells were cultured in DMEM high-glucose medium, and 4T1 cells in RPMI-1640 medium, both supplemented with 10 % FBS and 1 % antibiotic-antimycotic solution, maintained at 37 °C in a 5 % CO₂ incubator with 18–20 % O₂, and sub-cultured every 2–3 days. Cells at passage 59 (MCF7) and 24 (4T1) were used for cytotoxicity assessment. Cells were seeded into 96-well plates (5 × 10⁴ cells per well) and treated with six concentrations of Han: Nd³⁺ NPs (6.25, 12.5, 25, 50, 100, 200 µg/mL). Doxorubicin (1 µM) served as a positive control, while untreated cells (negative control) and a blank media control were included to ensure any cytotoxic effects were due to the test compounds. This systematic study design enables a comprehensive evaluation of Han: Nd³⁺ NPs as a potential therapeutic agent for breast cancer, with implications for dose optimization and targeted drug delivery strategies.

### 2.9 Statistical analysis

All experiments were performed in triplicate to ensure statistical robustness. Data analysis was conducted using GraphPad Prism 9.0 and relevant statistical software packages. Results are presented as mean ± standard deviation (SD). Statistical significance was assessed using one-way analysis of variance (ANOVA), followed by Tukey’s post hoc test for multiple comparisons. A significance threshold of *p* < 0.05 was considered statistically significant. The half-maximal inhibitory concentration (IC₅₀) values were determined using nonlinear regression (sigmoidal dose-response curve fitting) in GraphPad Prism 9.0.

## 3 Results and discussion

### 3.1 X-ray diffraction spectroscopy (XRD)

**Fig. 1:**
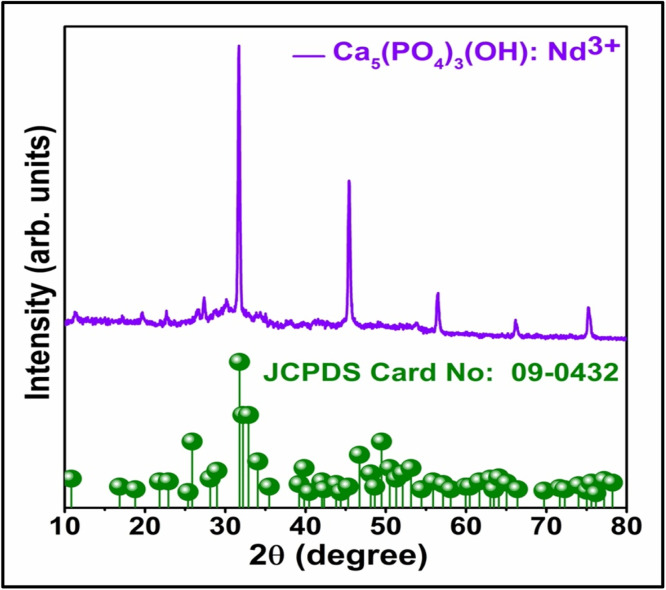
XRD spectrum of Han: Nd^3+^ NPs. XRD pattern of Han: Nd^3+^ NPs has the sharp and well-defined peaks correspond to the hexagonal phase of hydroxyapatite, as confirmed by comparison with the standard JCPDS card no. 09–0432 (green markers). The absence of secondary phase peaks indicates the successful incorporation of Nd³⁺ ions into the hydroxyapatite lattice without altering its crystallinity. This structural confirmation supports the phase purity and crystalline nature of the synthesized nanocomposites.

The XRD pattern of Han: Nd³⁺ NPs (Fig. 1) confirms the hexagonal phase of hydroxyapatite, matching JCPDS card No. 09–0432 and indicating successful neodymium substitution without lattice distortion. The diffraction peaks, FWHM values, and crystallite sizes (Table 1) reveal a well-defined structure with an average size of ∼26.6 nm, signifying high structural order essential for biomedical applications. This uniform crystallinity supports stable drug loading and release in breast cancer therapy, minimizing off-target effects [18]. Functionalization with specific ligands further enhances tumor selectivity [19], while the distinct optical properties of neodymium-doped NPs enable improved imaging and early tumor detection [20]. The crystallographic data also suggest favorable biocompatibility, underscoring Han: Nd³⁺ NPs suitability for safe, targeted *in vivo* applications.

**Table 1:** XRD peak positions, corresponding full width at half maximum (FWHM), and crystalline size of Han: Nd³⁺ NPs.

Peak Position (2 Theta)	FWHM	Crystalline Size (nm)
31.72	0.246	26.793
45.49	0.233	31.397
56.42	0.291	26.342
66.23	0.313	23.012
75.17	0.205	25.729
Average: 26.655		

### 3.2 Hydrodynamic size and zeta potential analysis

**Fig. 2:**
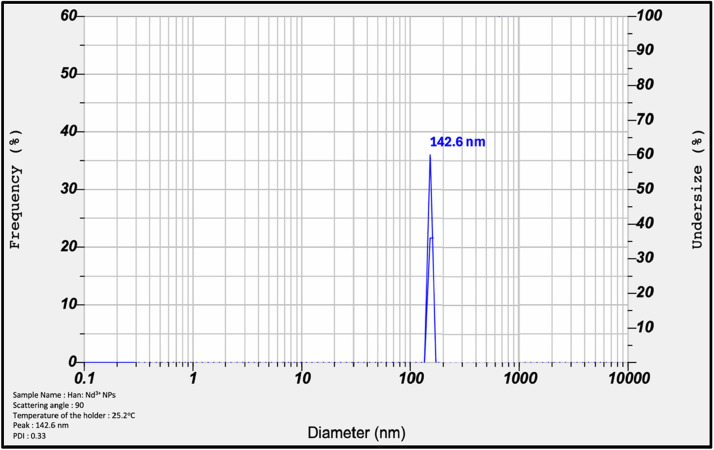
Dynamic Light Scattering (DLS) Analysis of Han: Nd³⁺ NPs. The particle size distribution profile shows a sharp, unimodal peak centered around 142.6 nm, indicating a relatively narrow size distribution. The data reflect a polydispersity index (PDI) of 0.33, suggesting moderate monodispersity and acceptable uniformity for biological applications. The x-axis is plotted on a logarithmic scale, and the primary y-axis (left) indicates frequency ( %), while the secondary y-axis (right) represents the cumulative undersize ( %) distribution. This hydrodynamic diameter measurement confirms the colloidal stability and dispersibility of the nanocomposites in aqueous media.

**Fig. 3:**
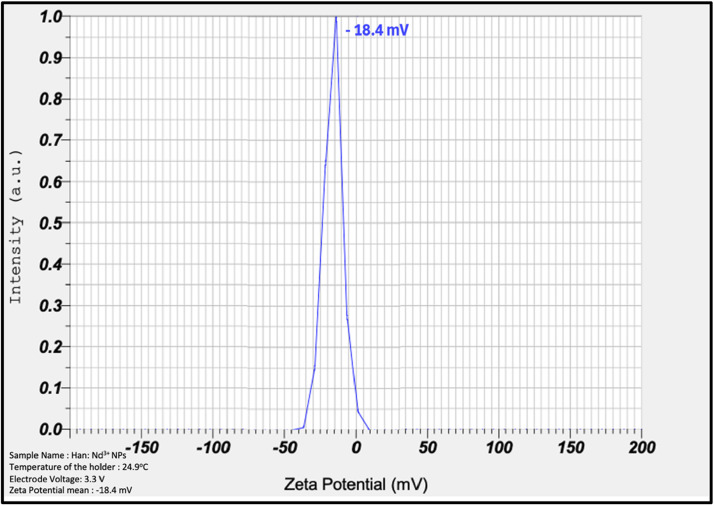
Zeta Potential Analysis of Han: Nd³⁺ NPs. Zeta potential measurements demonstrate a sharp, narrow peak centered around −18.4 mV. The negative surface charge signifies moderate electrostatic repulsion between particles, which contributes to colloidal stability and reduces the likelihood of aggregation. The graph illustrates intensity (a.u.) on the y-axis and zeta potential (mV) on the x-axis. The observed negative charge is conducive to good aqueous dispersibility and minimal nonspecific protein adsorption, enhancing the biocompatibility of the nanocomposites in biological systems.

The hydrodynamic diameter and surface charge of the synthesized Han: Nd^3+^ NPs were assessed to determine their colloidal stability and appropriateness for biological applications. DLS tests indicated a singular, narrow peak at approximately 142.6 nm, accompanied by a polydispersity index (PDI) of 0.33, signifying a fairly monodisperse nanoparticle population (Fig. 2). The DLS profile closely corresponds with the particle size distribution characteristic of stable colloidal systems, which are advantageous for cellular absorption and consistent *in vitro* interactions [27]. Furthermore, zeta potential analysis revealed a value of −18.4 mV, indicating moderate electrostatic repulsion among particles (Fig. 3), hence contributing to the maintenance of colloidal stability in suspension. The physicochemical qualities are essential for assessing nanomaterial behavior in biological contexts since aggregation or instability may result in variations in cellular responses or toxicity results [28]. The observed hydrodynamic size range is suitable for promoting endocytosis, whereas the negative surface charge diminishes the probability of nonspecific protein adsorption and improves dispersion in aqueous biological environments [41]. The results substantiate the application of Han: Nd^3+^ NPs *in vitro* cytotoxicity investigations and prospective diagnostic use, where uniform nanoparticle behavior in the exposure medium is crucial for reproducibility and interpretation of biological effects.

### 3.3 X-ray photoelectron spectroscopy (XPS)

**Fig. 4:**
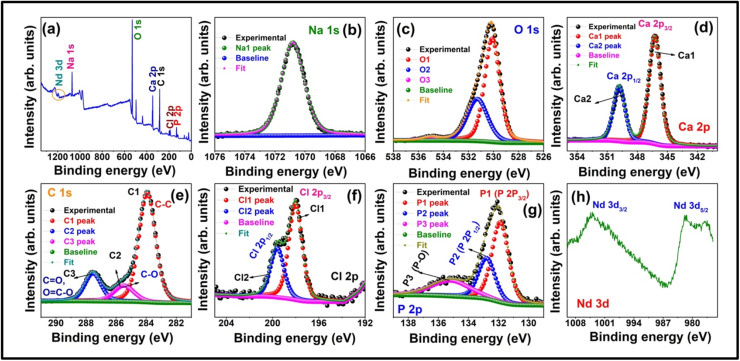
X-ray photoelectron spectroscopy (XPS) analysis of Han: Nd³⁺ NPs. (a) Spectrum confirming the presence of elemental peaks including Nd 3d, Na 1 s, O 1 s, Ca 2p, P 2p, Cl 2p, and C 1 s. (b) High-resolution XPS spectrum of Na 1 s shows the binding energy profile with fitted Na peaks and baseline. (c) O 1 s spectrum deconvoluted into three oxygen species: O1 (lattice oxygen), O2 (hydroxyl groups), and O3 (adsorbed water), indicating the typical oxygen environment in HA structures. (d) Ca 2p spectrum showing characteristic Ca 2p₃/₂ and Ca 2p₁/₂ peaks, confirming the divalent calcium state in the HA matrix. (e) C 1 s spectrum displaying three components: C1 (C–C/C–H), C2 (C–O/C=O), and C3 (carbonate contaminant or surface species), included as reference for binding correction. (f) Cl 2p doublet peaks (Cl 2p₁/₂ and Cl 2p₃/₂) representing trace residuals from precursors or processing. (g) P 2p spectrum deconvoluted into P1, P2, and P3 components reflecting phosphate environments typical of hydroxyapatite. (h) Han: Nd³⁺ NPs 3d spectrum revealing Nd 3d₅/₂ and Nd 3d₃/₂ peaks, verifying the successful doping of neodymium into the HA matrix. These spectral profiles validate the elemental composition, chemical states, and successful integration of Nd³⁺ within the hydroxyapatite lattice.

The XPS survey spectrum of Han: Nd³⁺ NPs reveals their elemental composition and chemical states, crucial for their potential in breast cancer treatment and diagnostics. The XPS scan spectrum of Han: Nd³⁺ NPs validated the presence of essential components, including Nd, P, Ca, O, and trace amounts of Na and Cl (Fig. 4). Significantly, Nd³⁺ signals were observed at binding energies of 1003.98 eV (Nd 3d_3/2_) and 981.35 eV (Nd 3d_5/2_), confirming the successful incorporation of trivalent ions into the HA lattice. The peaks of phosphorus (P 2p), calcium (Ca 2p), and oxygen (O1s) aligned with conventional hydroxyapatite signatures, confirming structural integrity. Signals of sodium and chlorine may be ascribed to synthesis precursors or ambient exposure. These compositional findings corroborate the effective doping and structural appropriateness of Han: Nd³⁺ NPs for biomedical applications.

### 3.4 Scanning electron microscopy with EDaX (SEM-EDaX)

**Fig. 5:**
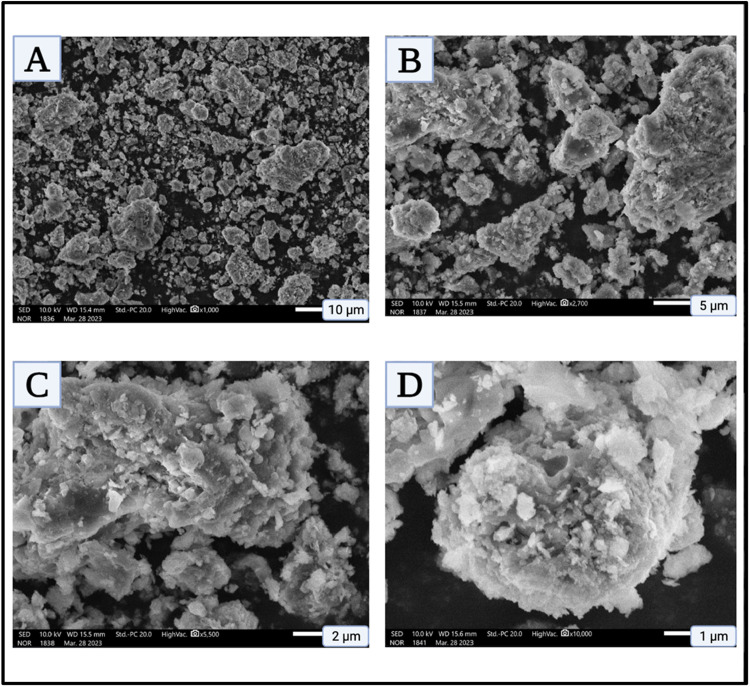
Scanning electron microscopy (SEM) images of Han: Nd³⁺ NPs at increasing magnifications. (A) Low magnification image (1000 ×) displaying widespread distribution and aggregate formation of Han: Nd³⁺ NPs over a broad field. (B) Intermediate magnification (2700 ×) highlighting loosely packed, irregularly shaped nanoparticle clusters. (C) Higher magnification (5500 ×) revealing granular and flaky surface morphologies indicative of anisotropic crystal growth. (D) Ultra-high magnification (10,000 ×) showing dense, spherical agglomerates with porous surface textures, possibly promoting cellular uptake. All images confirm the microstructural characteristics of Han: Nd³⁺ NPs, including size uniformity, particle roughness, and agglomeration tendency. Scale bars: 10 μm (A), 5 μm (B), 2 μm (C), and 1 μm (D).

**Fig. 6:**
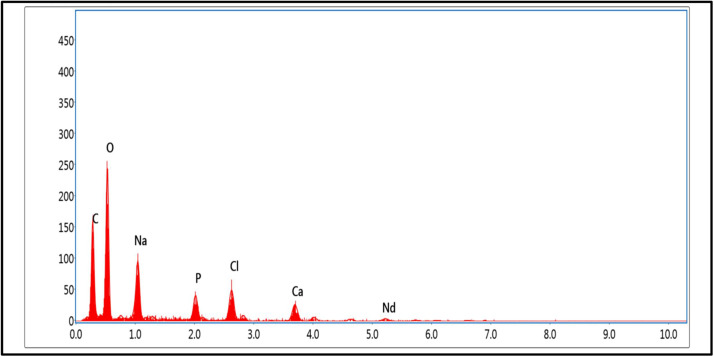
Energy-dispersive X-ray spectroscopy spectrum of Han: Nd³⁺ NPs. The spectrum confirms the elemental composition of the synthesized nanocomposite, with characteristic peaks for oxygen (O), carbon (C), sodium (Na), phosphorus (P), calcium (Ca), chlorine (Cl), and the dopant neodymium (Nd). The dominant O and Ca peaks reflect the hydroxyapatite matrix, while the presence of P and Na is consistent with lattice constituents. The minor Nd peak validates successful incorporation of neodymium ions within the HA structure. Trace Cl may be attributed to precursor residues. This analysis confirms the elemental purity and successful doping essential for intended biomedical applications.

The SEM analysis (Fig. 5) of Han: Nd³⁺ NPs reveals a well-defined crystalline structure with some degree of aggregation, with the average particle size estimated at approximately 28 ± 4 nm, a common occurrence in hydroxyapatite-based materials due to interparticle interactions or synthesis conditions. This morphology aligns with the expected characteristics of hydroxyapatite-derived nanoparticles, reinforcing their potential biomedical applications. EDX analysis confirms the presence of carbon (C), oxygen (O), chlorine (Cl), calcium (Ca), phosphorus (P), sodium (Na), and neodymium (Nd) (Fig. 6), consistent with CHN analysis and complexometric titration results (Table 2). Elemental composition analysis indicates carbon (19.07 %) and oxygen (24.45 %) as predominant elements, likely from organic moieties introduced during synthesis or surface modifications enhancing biocompatibility. Sodium (11.37 %) may originate from precursor chemicals, influencing solubility and surface charge, thus impacting drug delivery suitability [[22], [23]]. Phosphorus (6.22 %) and calcium (12.96 %) confirm the hydroxyapatite framework, ensuring structural stability and biocompatibility [24]. Neodymium (14.74 %), though present in lower amounts, imparts unique luminescent properties applicable to imaging and diagnostics in oncology and regenerative medicine. Its incorporation into the hydroxyapatite matrix enhances structural stability and expands multifunctional applications, including targeted drug delivery and bioimaging. These findings confirm the successful synthesis of Han: Nd^3+^ NPs with well-defined morphology and tailored elemental composition, supporting their potential in cancer therapy, imaging, and bone tissue engineering.

**Table 2:** Percentage analytical data of synthesized Han: Nd^3+^ NPs.

Element	Weight ( % found)	Atomic weight ( % found)	Error ( % found)
C	19.07	34.88	13.23
O	24.45	33.57	10.76
Na	11.37	10.86	10.62
P	6.22	4.41	10.4
Cl	11.16	6.92	8.26
Ca	12.96	7.11	9.94
Nd	14.74	2.25	18.11

### 3.5 *In vitro* cytotoxicity studies

**Fig. 7:**
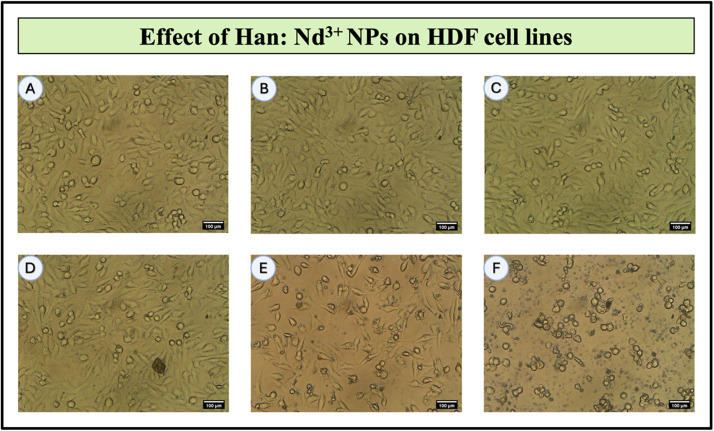
Bright-field microscopic images showing the dose-dependent effect of Han: Nd³⁺ nanoparticles (NPs) on human dermal fibroblast (HDF) cell morphology after 24 h of treatment. (A) 6.25 μg/mL; (B) 12.5 µg/mL; (C) 25 µg/mL; (D) 50 µg/mL; (E) 100 µg/mL; (F) 200 µg/mL. Cells were cultured under standard conditions and exposed to increasing concentrations of Han:Nd³⁺ NPs. At lower concentrations (B–D), HDF cells retained their elongated spindle-like morphology, suggesting minimal cytotoxic impact. However, at higher concentrations (E–F), cells exhibited morphological alterations such as rounding, shrinkage, and detachment—indicative of compromised membrane integrity and reduced viability. The dose-responsive morphological changes align with MTT assay results and underscore the importance of concentration-dependent cytocompatibility in evaluating nanomaterials for biomedical applications. All scale bars represent 100 µm.

**Fig. 8:**
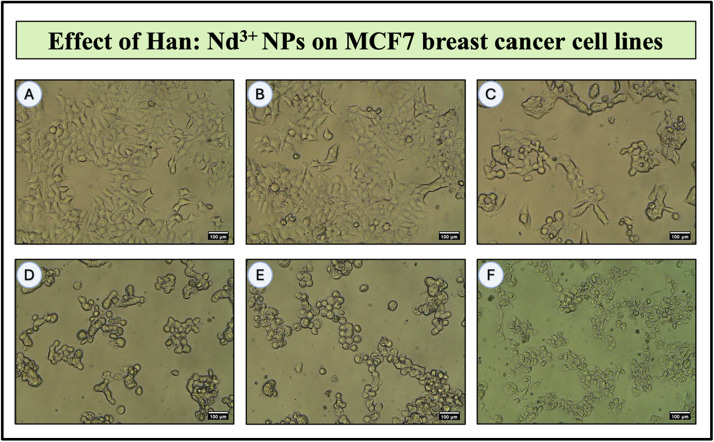
Bright-field microscopic images illustrating the dose-dependent cytotoxic effects of Han: Nd³⁺ nanoparticles (NPs) on MCF7 human breast cancer cell lines after 24 h of treatment. (A) 6.25 μg/mL; (B) 12.5 µg/mL; (C) 25 µg/mL; (D) 50 µg/mL; (E) 100 µg/mL; (F) 200 µg/mL. MCF7 cells exposed to increasing concentrations of Han:Nd³⁺ NPs exhibited clear morphological alterations. Untreated cells maintained a healthy, epithelial-like adherent morphology. As concentration increased (B–F), cells displayed progressive signs of cytotoxicity including loss of adherence, cell shrinkage, rounding, and membrane blebbing. At the highest concentrations (E and F), extensive cell detachment and death were observed, suggesting necrotic or apoptotic pathways. These visual observations are consistent with MTT assay results, validating the concentration-dependent anticancer potential of the synthesized nanoparticles. All images were captured at 100 µm scale.

**Fig. 9:**
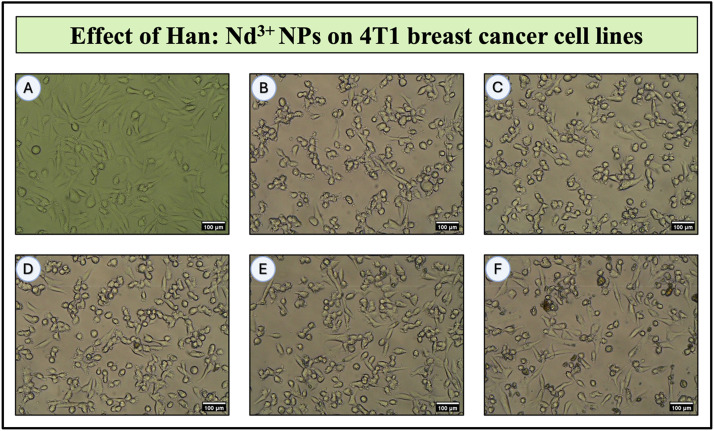
Bright-field microscopic images showing the effect of Han: Nd³⁺ nanoparticles (NPs) on 4T1 murine breast cancer cell lines after 24 h of exposure. (A) 6.25 μg/mL; (B) 12.5 µg/mL; (C) 25 µg/mL; (D) 50 µg/mL; (E) 100 µg/mL; (F) 200 µg/mL. Untreated 4T1 cells (A) exhibited characteristic fibroblast-like morphology with dense and healthy adherence. Upon treatment with Han:Nd³⁺ NPs (B–F), there was a clear dose-dependent cytotoxic response. At lower concentrations (B–C), early morphological signs of cytotoxicity such as rounding and slight detachment were observed. As the dose increased (D–F), extensive cell shrinkage, membrane damage, and loss of monolayer integrity became prominent. The most significant cytotoxic effects occurred at 100 and 200 µg/mL, consistent with the MTT assay results. These visual findings support the dose-responsive anticancer potential of Han:Nd³⁺ NPs in murine breast cancer models. All images were taken at 100 µm scale.

To investigate the biocompatibility of Han: Nd^3+^ NPs with normal human cells, human dermal fibroblasts (HDF) were subjected to various concentrations (6.25 to 200 µg/mL) for 24 h, and their vitality was evaluated using the MTT assay. The proportion of viable cells diminished progressively with elevated nanoparticle concentrations, signifying a dose-dependent cytotoxic response (Table 3; Fig. 7, Fig. 8, Fig. 9). At the least quantity of 6.25 µg/mL, cell viability was maintained at 76.67 %, signifying little cytotoxic effects. With increasing concentration, a progressive decline in cell viability was noted, with recorded viabilities of 69.89 %, 63.85 %, and 57.53 % at 12.5, 25, and 50 µg/mL, respectively. Significant reductions were observed at elevated concentrations: 46.05 % at 100 µg/mL and 35.63 % at 200 µg/mL. The IC₅₀ value, derived from the dose-response curve, was around 71.78 µg/mL, signifying significant cytotoxicity in normal cells.

**Table 3:** Cell viability of Han: Nd³⁺ NPs on HDF (normal), MCF-7 (breast cancer), and 4T1 (mouse mammary carcinoma) cell lines after 24-hour incubation, assessed via MTT assay. Values are expressed as mean ± standard deviation from two independent experiments. Superscript letters (a–h) indicate statistically significant differences within each cell line column, as determined by one-way ANOVA followed by Tukey’s multiple comparisons test (*p* < 0.05). Groups not sharing a common superscript differ significantly.

Concentration (µg/ml)	Normal Cells (HDF)	MCF7 Cells	4T1 Cells
Untreated	100.00 ± 0.02^h^	100.00 ± 0.01^h^	100.00 ± 0.02^h^
Dox-1µM	35.90 ± 0.02^g^	51.64 ± 0.06^g^	46.25 ± 0.02^g^
6.25	76.67 ± 0.04^f^	87.79 ± 0.02^f^	90.16 ± 0.04^f^
12.5	69.89 ± 0.01^b^	79.34 ± 0.01^b^	76.23 ± 0.03^b^
25	63.85 ± 0.02^d^	63.21 ± 0.05^d^	72.18 ± 0.00^d^
50	57.53 ± 0.09^e^	41.73 ± 0.01^e^	64.57 ± 0.05^e^
100	46.05 ± 0.10^a^	28.95 ± 0.01^a^	45.69 ± 0.05^a^
200	35.63 ± 0.00^c^	2.16 ± 0.01^c^	34.97 ± 0.05^c^

**Fig. 10:**
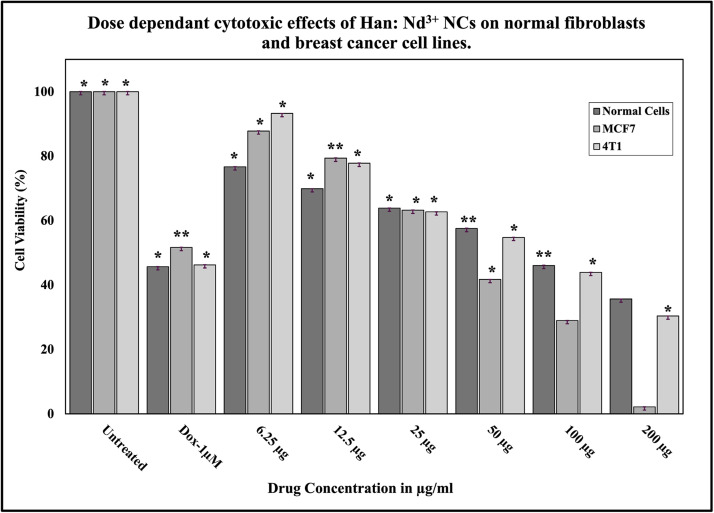
Dose-dependent cytotoxicity evaluation of Han: Nd³⁺ NPs in human breast cancer cell lines (MCF7 and 4T1) and normal human dermal fibroblast (HDF) cells. Cells were treated with increasing concentrations (6.25 to 200 µg/mL) of Han: Nd³⁺ NPs for 24 h, and viability was assessed via the MTT assay. Untreated cells served as the 100 % viability control, and Doxorubicin (1 µM) was used as a positive control for cytotoxicity. Cell viability is presented as mean ± standard deviation (*n* = 2). A significant dose-dependent reduction in viability was observed in both cancer cell lines, with 200 µg/mL inducing the strongest cytotoxicity, particularly in MCF7 cells. Normal cells exhibited relatively higher viability across concentrations, suggesting selective toxicity. Statistical significance was determined using one-way ANOVA followed by Tukey’s post hoc test. *indicates *p* < 0.05 and ** indicates *p* < 0.01 compared to the untreated control group within each cell line.

Fig. 8, Fig. 9 illustrate the dose-dependent cytotoxic effects of Han: Nd³⁺ NPs on MCF7 and 4T1 breast cancer cells at concentrations ranging from 6.25 to 200 µg/mL. MTT assay results revealed a significant reduction in cell viability, with MCF7 cells exhibiting greater sensitivity (IC50 = 36.2 µg/mL) than 4T1 cells (IC50 = 64.4 µg/mL). One-way ANOVA confirmed a statistically significant decrease in viability (*p* < 0.05) at concentrations ≥50 µg/mL, highlighting the cytotoxic potential of Han: Nd³⁺ NPs. At concentration 200 µg/mL, Han: Nd³⁺ NPs reduced MCF7 cell viability to 2.16 %, closely approaching the effect of doxorubicin (51.64 % at 1 µM). In contrast, 4T1 cells retained 30.37 % viability at 200 µg/mL, indicating a differential response likely due to variations in cellular uptake and resistance mechanisms. Dose-response analysis (Fig. 10) showed significant cytotoxicity (*p* < 0.05) at ≥25 µg/mL for MCF7 and ≥50 µg/mL for 4T1. At higher concentrations, Han: Nd³⁺ NPs exhibited comparable cytotoxicity to doxorubicin (*p* > 0.05). The results demonstrate that Han: Nd³⁺ NPs exhibit strong and selective cytotoxicity against MCF7 and 4T1 breast cancer cells, while maintaining notable biocompatibility at lower concentrations in normal fibroblasts. This differential response underscores a promising therapeutic window that can be further optimized through surface engineering or targeted delivery strategies to enhance efficacy while minimizing off-target effects [29].

## 4 Discussion

The meticulous characterization and cytotoxicity assessment of Han: Nd³⁺ NPs reveal encouraging attributes for biomedical applications. Given the natural affinity of hydroxyapatite for bone tissue, there is a potential risk of unintended accumulation and integration into the skeletal system. To address this, future work will focus on developing protective surface coatings and specific ligands like folate, HER2 antibodies to enhance active targeting to control release and direct biodistribution. These strategies aim to limit off-target deposition and enable renal clearance, thereby reducing cumulative toxicity*. In vivo* studies will be essential to further evaluate safety, biodistribution, and long-term effects. An XRD investigation validated the hexagonal crystal structure of hydroxyapatite, which is devoid of secondary phases, signifying effective neodymium doping without structural degradation. The average crystallite size, calculated using the Debye-Scherrer equation, aligned with nanoscale dimensions, is essential for biological compatibility. XPS confirmed the chemical state and successful integration of Nd³⁺ within the HA lattice, emphasizing interactions that could affect surface reactivity and biological response. While the current study relies on the passive Enhanced Permeability and Retention (EPR) effect for tumor accumulation, future investigations will explore functionalizing Han: Nd³⁺ NPs with tumor-specific ligands like folate, HER2 antibodies to enhance active targeting. Ligand-mediated delivery has been shown to improve tumor selectivity, intracellular uptake, and therapeutic precision compared to EPR alone, particularly in poorly vascularized or heterogeneous tumors [42]. At the same time, energy dispersive X-ray analysis validated the elemental composition and uniform distribution of Ca, P, O, and Nd.

Dynamic light scattering (DLS) measurements revealed a hydrodynamic diameter of 142.6 nm and a polydispersity index (PDI) of 0.33. The specified values are within the ideal size range for cellular uptake through endocytosis, as particles under 200 nm are recognized to enhance intracellular internalization, especially via clathrin-mediated mechanisms [30,31]. Moderate PDI indicates sufficient uniformity is required for consistent biological results. Zeta potential measurement indicated a surface charge of −18.4 mV, signifying adequate electrostatic repulsion to avert particle aggregation, essential in serum-rich biological contexts [32]. Metastable systems exhibiting zeta potentials ranging from −10 mV to −30 mV are deemed optimal for sustaining dispersion and minimizing nonspecific interactions [33]. Due to its chemical resemblance to bone mineral, hydroxyapatite's intrinsic attraction for bone may pose a potential risk of unintended skeletal buildup [34]. This issue is reflected in pharmaceutics literature that emphasizes the dual potential of HA in targeted drug delivery and inadvertent biodistribution [21]. Consequently, forthcoming research will focus on integrating surface functionalization using folate, or HER2 ligands and biodegradable polymeric coatings, chitosan, to improve active tumor targeting, regulate drug release, and facilitate renal clearance [35].

The cytotoxicity data indicated a considerable dose-dependent decrease in the viability of MCF7 and 4T1 cancer cells, with relatively minimal effects on HDF normal cells. This selective cytotoxicity holds promise for therapeutic applications, consistent with prior studies on rare-earth-doped HA nanocomposites [36,37]. Compared to the conventional chemotherapeutic drug doxorubicin, Han: Nd³⁺ nanoparticles exhibited reduced cytotoxicity in both cell types. In MCF7 cells, doxorubicin (1 µM) diminished viability to 51.64 %, but Han: Nd³⁺ NPs at 100 µg/mL further decreased viability to 28.95 % and a mere 2.16 % at 200 µg/mL. A comparable pattern was noted in 4T1 cells when doxorubicin diminished viability to 46.25 %, in contrast to 30.37 % at the maximum measured concentration of Han: Nd³⁺ NPs. In contrast to doxorubicin, Han: Nd³⁺ NPs are anticipated to have enhanced structural compatibility, reduced systemic toxicity, and adjustable functioning. Doxorubicin exerts its effects via DNA intercalation and topoisomerase II inhibition, whereas the cytotoxicity of Han: Nd³⁺ NPs is presumably attributable to surface interactions, oxidative stress, and ion exchange mechanisms, providing an alternative pathway for the suppression of cancer cells. This shows that although Han: Nd³⁺ NPs may not approach doxorubicin in efficacy, they provide potential as a complementary or alternative therapeutic platform that might be enhanced for targeted distribution and combined therapy regimens.

Subsequent investigations will incorporate reactive oxygen species assays and apoptosis indicators to validate the mechanistic discoveries. Furthermore, the application of tri-sodium citrate as a stabilizing agent during synthesis seems to enhance dispersion and diminish aggregation, hence promoting colloidal stability and uniform *in vitro* performance [38]. The nanoparticles demonstrated marginally diminished cytotoxic efficiency compared to doxorubicin, a recognized chemotherapeutic drug, suggesting decreased nonspecific toxicity a beneficial attribute for nanotherapeutics [39]. Significantly, these nanocomposites maintain structural integrity and imaging compatibility, indicating their potential as multifunctional theranostic agents [40]. Their adaptability may be augmented through surface engineering to boost tumor selectivity and therapeutic index. The physicochemical characteristics, *in vitro* cytotoxicity, and structural integrity of Han: Nd³⁺ NPs endorse their potential as targeted and diagnostic agents.

## 5 Conclusion

This study underscores the great potential of Han: Nd³⁺ nanoparticles as multifunctional agents for breast cancer diagnosis and treatment. XRD proved the production of a pure, crystalline hydroxyapatite phase. In contrast, XPS validated the successful incorporation of neodymium ions into the lattice without affecting material integrity. SEM morphological characterisation demonstrated consistent particle production, while EDX validated elemental composition. The hydrodynamic size (∼142.6 nm), moderate polydispersity index (0.33), and zeta potential (−18.4 mV) exhibited advantageous physicochemical characteristics for biological interactions and colloidal stability. The nanoparticles demonstrated dose-dependent cytotoxicity in MCF7 and 4T1 breast cancer cells while exhibiting somewhat lesser toxicity in normal HDF fibroblasts, suggesting a potential therapeutic window. Despite *in vitro* evidence indicating selective cytotoxic efficacy, additional development is necessary to improve specificity and augment therapeutic relevance. These Han: Nd^3+^ NPs demonstrate cohesive structural stability and anticancer efficacy, affirming their use in theranostic applications, including drug transport and imaging. Future research should concentrate on *in vivo* biodistribution and pharmacokinetic investigations, surface modification for active targeting, and validation in pertinent animal tumour models to advance translational use. These steps are crucial for assessing safety, selective accumulation, and therapeutic efficacy before clinical application.

## Funding

The authors declare that there is no financial support from external sources.

## CRediT authorship contribution statement

**K.Sai Manogna:** Writing – original draft, Methodology, Formal analysis, Data curation. **K. Kusuma:** Data curation. **G.Rajasekhara Reddy:** Data curation. **B.Deva Prasad Raju:** Writing – review & editing, Methodology, Conceptualization. **N.John Sushma:** Writing – review & editing, Methodology, Formal analysis, Conceptualization.

## Declaration of competing interest

The authors declare the following financial interests/personal relationships which may be considered as potential competing interests:

The authors declare no conflict of interest If there are other authors, they declare that they have no known competing financial interests or personal relationships that could have appeared to influence the work reported in this paper.

## Data Availability

No data was used for the research described in the article.
